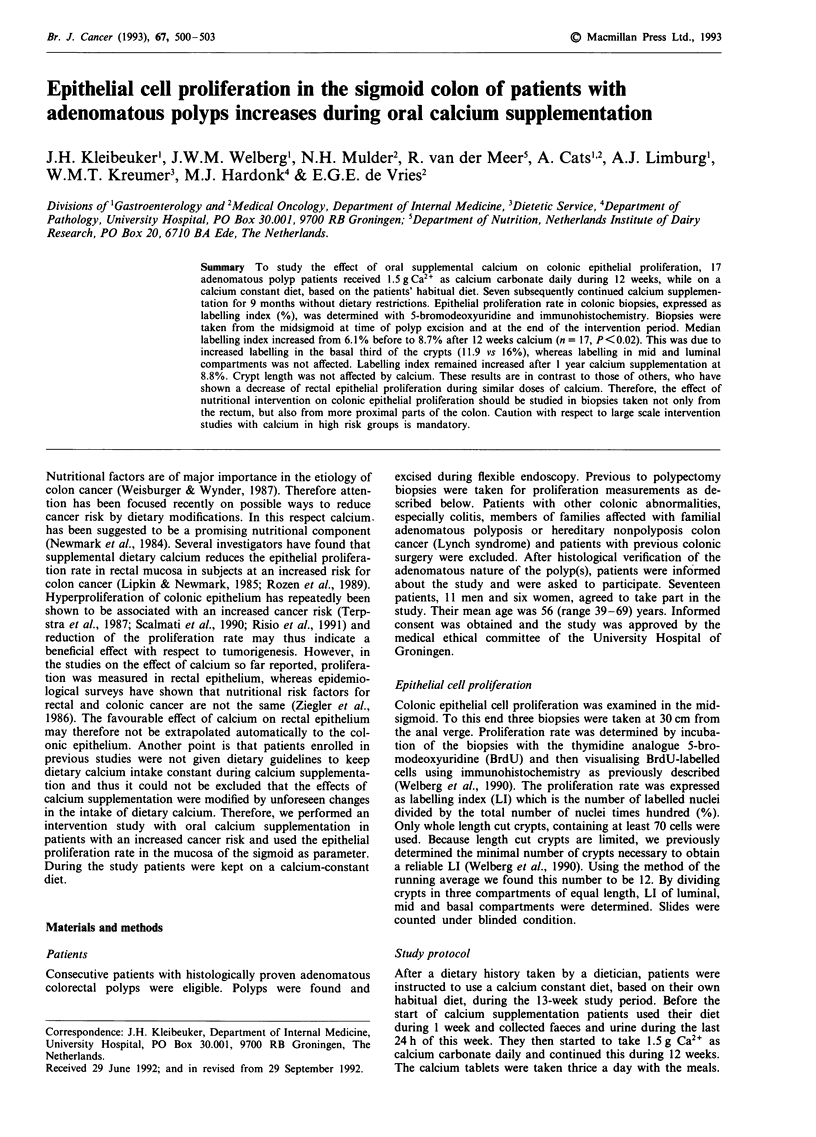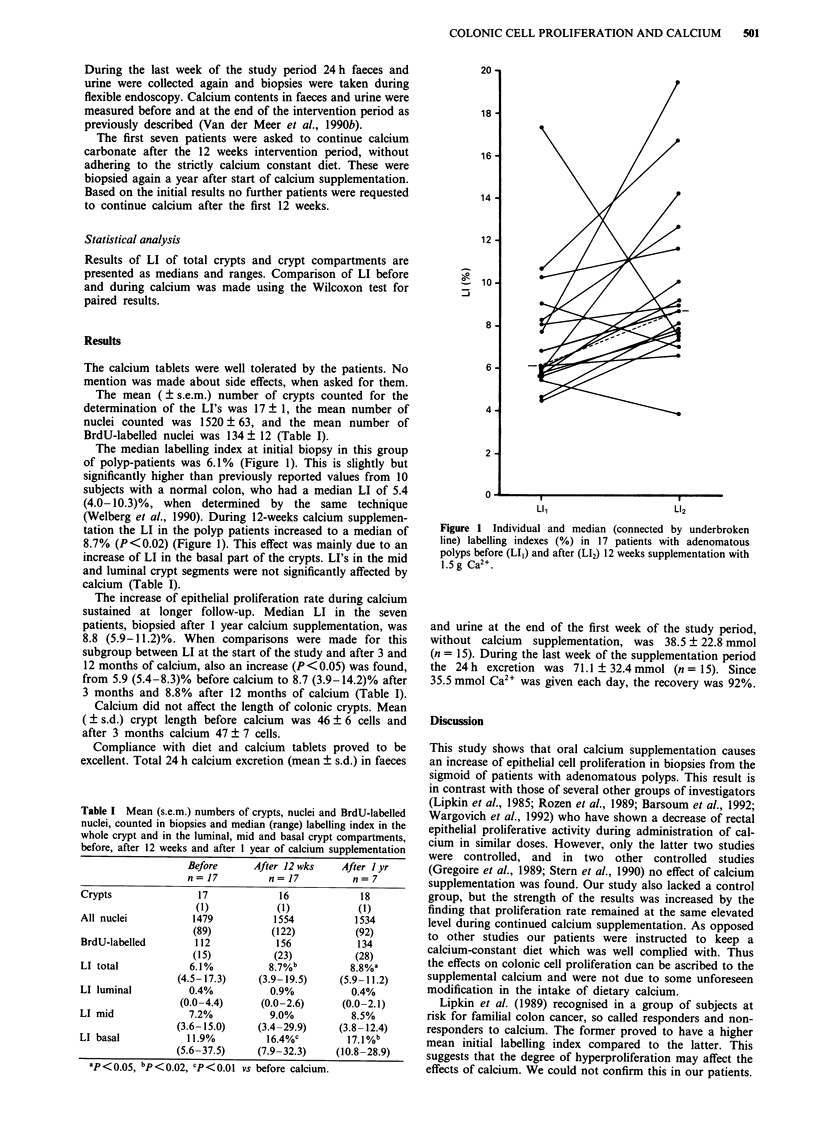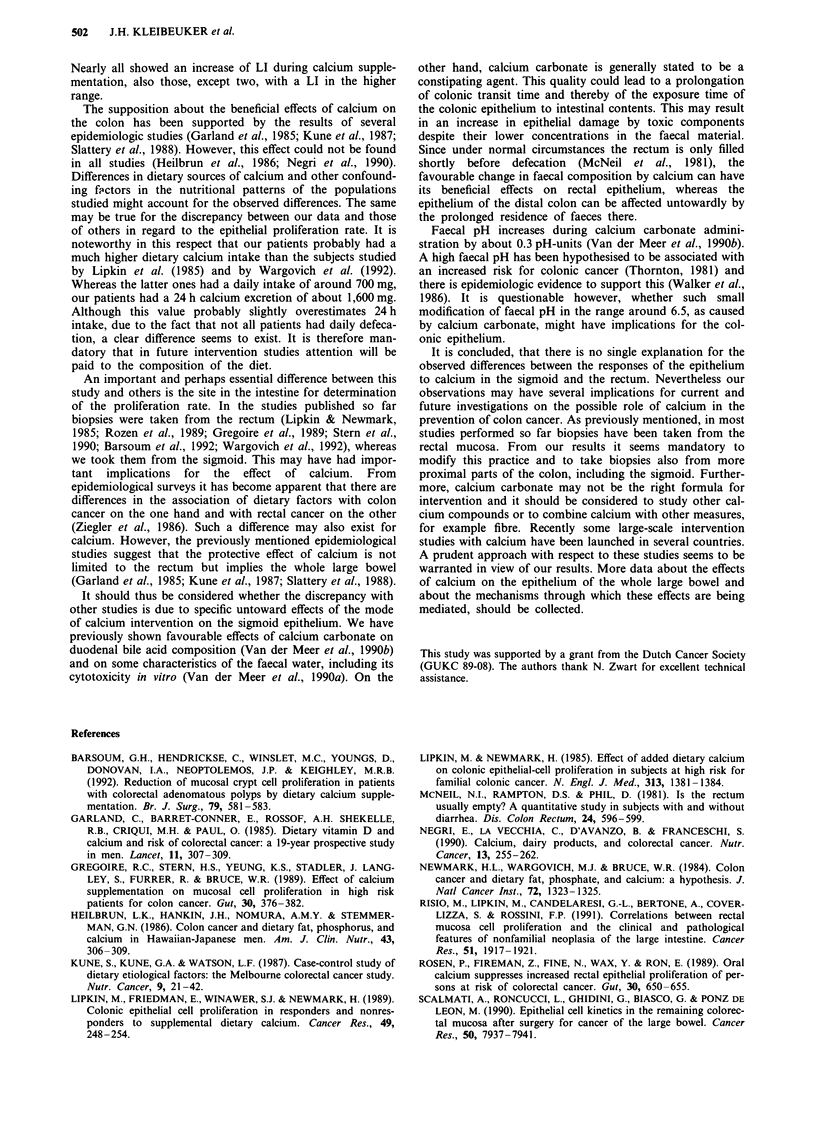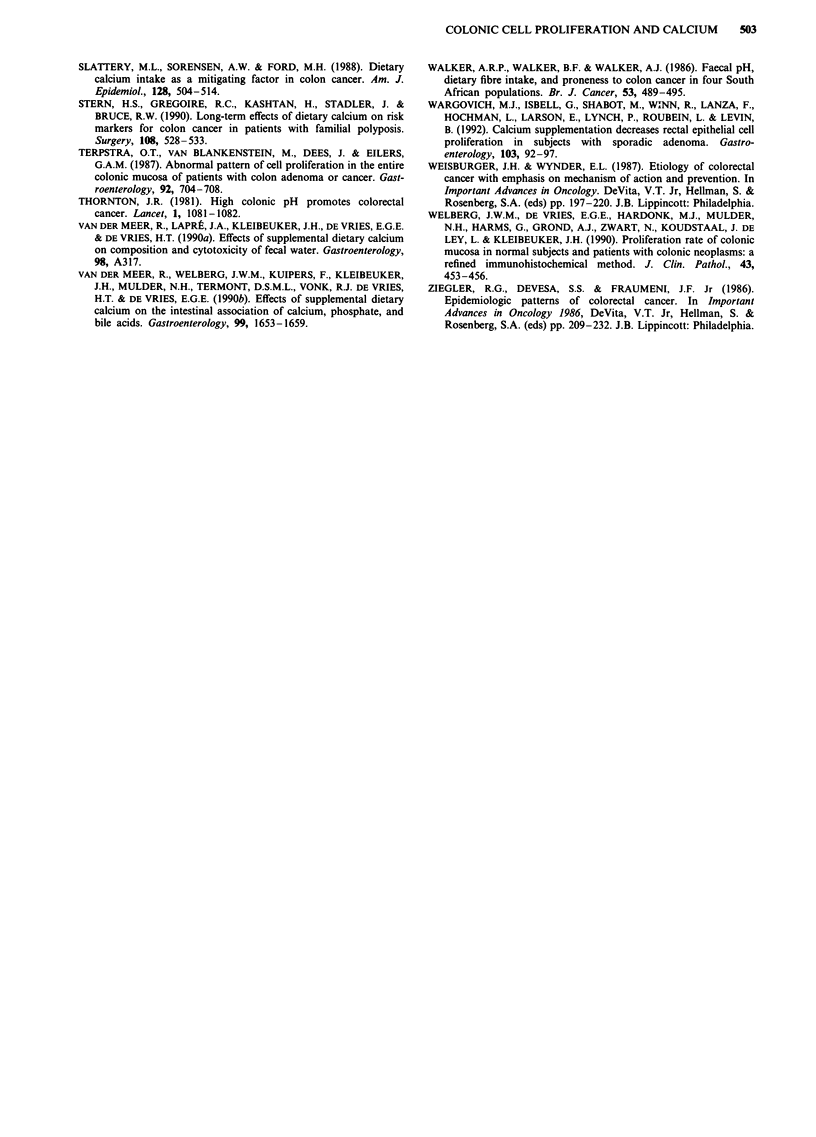# Epithelial cell proliferation in the sigmoid colon of patients with adenomatous polyps increases during oral calcium supplementation.

**DOI:** 10.1038/bjc.1993.93

**Published:** 1993-03

**Authors:** J. H. Kleibeuker, J. W. Welberg, N. H. Mulder, R. van der Meer, A. Cats, A. J. Limburg, W. M. Kreumer, M. J. Hardonk, E. G. de Vries

**Affiliations:** Division of Gastroenterology, University Hospital, Groningen, The Netherlands.

## Abstract

To study the effect of oral supplemental calcium on colonic epithelial proliferation, 17 adenomatous polyp patients received 1.5 g Ca2+ as calcium carbonate daily during 12 weeks, while on a calcium constant diet, based on the patients' habitual diet. Seven subsequently continued calcium supplementation for 9 months without dietary restrictions. Epithelial proliferation rate in colonic biopsies, expressed as labelling index (%), was determined with 5-bromodeoxyuridine and immunohistochemistry. Biopsies were taken from the midsigmoid at time of polyp excision and at the end of the intervention period. Median labelling index increased from 6.1% before to 8.7% after 12 weeks calcium (n = 17, P < 0.02). This was due to increased labelling in the basal third of the crypts (11.9 vs 16%), whereas labelling in mid and luminal compartments was not affected. Labelling index remained increased after 1 year calcium supplementation at 8.8%. Crypt length was not affected by calcium. These results are in contrast to those of others, who have shown a decrease of rectal epithelial proliferation during similar doses of calcium. Therefore, the effect of nutritional intervention on colonic epithelial proliferation should be studied in biopsies taken not only from the rectum, but also from more proximal parts of the colon. Caution with respect to large scale intervention studies with calcium in high risk groups is mandatory.


Br. J. Cancer (1993), 67, 500-503

© Macmillan Press Ltd., 1993

### Epithelial cell proliferation in the sigmoid colon of patients with adenomatous polyps increases during oral calcium supplementation

J.H. Kleibeuker[1], J.W.M. Welberg[1], N.H. Mulder[2], R. van der Meer[5], A. Cats[1,2], A.J. Limburg[1], W.M.T. Kreumer[3], M.J. Hardonk[4] & E.G.E. de Vries[2]

*Divisions of [1]Gastroenterology and [2]Medical Oncology, Department of Internal Medicine, [3]Dietetic Service, [4]Department of Pathology, University Hospital, PO Box 30.001, 9700 RB Groningen; [5]Department of Nutrition, Netherlands Institute of Dairy Research, PO Box 20, 6710 BA Ede, The Netherlands.*

**Summary** To study the effect of oral supplemental calcium on colonic epithelial proliferation, 17 adenomatous polyp patients received 1.5 g $Ca^{2+}$ as calcium carbonate daily during 12 weeks, while on a calcium constant diet, based on the patients' habitual diet. Seven subsequently continued calcium supplementation for 9 months without dietary restrictions. Epithelial proliferation rate in colonic biopsies, expressed as labelling index (%), was determined with 5-bromodeoxyuridine and immunohistochemistry. Biopsies were taken from the midsigmoid at time of polyp excision and at the end of the intervention period. Median labelling index increased from 6.1% before to 8.7% after 12 weeks calcium ($n = 17$, $P < 0.02$). This was due to increased labelling in the basal third of the crypts (11.9 *vs* 16%), whereas labelling in mid and luminal compartments was not affected. Labelling index remained increased after 1 year calcium supplementation at 8.8%. Crypt length was not affected by calcium. These results are in contrast to those of others, who have shown a decrease of rectal epithelial proliferation during similar doses of calcium. Therefore, the effect of nutritional intervention on colonic epithelial proliferation should be studied in biopsies taken not only from the rectum, but also from more proximal parts of the colon. Caution with respect to large scale intervention studies with calcium in high risk groups is mandatory.

Nutritional factors are of major importance in the etiology of colon cancer (Weisburger & Wynder, 1987). Therefore attention has been focused recently on possible ways to reduce cancer risk by dietary modifications. In this respect calcium has been suggested to be a promising nutritional component (Newmark *et al.*, 1984). Several investigators have found that supplemental dietary calcium reduces the epithelial proliferation rate in rectal mucosa in subjects at an increased risk for colon cancer (Lipkin & Newmark, 1985; Rozen *et al.*, 1989). Hyperproliferation of colonic epithelium has repeatedly been shown to be associated with an increased cancer risk (Terpstra *et al.*, 1987; Scalmati *et al.*, 1990; Risio *et al.*, 1991) and reduction of the proliferation rate may thus indicate a beneficial effect with respect to tumorigenesis. However, in the studies on the effect of calcium so far reported, proliferation was measured in rectal epithelium, whereas epidemiological surveys have shown that nutritional risk factors for rectal and colonic cancer are not the same (Ziegler *et al.*, 1986). The favourable effect of calcium on rectal epithelium may therefore not be extrapolated automatically to the colonic epithelium. Another point is that patients enrolled in previous studies were not given dietary guidelines to keep dietary calcium intake constant during calcium supplementation and thus it could not be excluded that the effects of calcium supplementation were modified by unforeseen changes in the intake of dietary calcium. Therefore, we performed an intervention study with oral calcium supplementation in patients with an increased cancer risk and used the epithelial proliferation rate in the mucosa of the sigmoid as parameter. During the study patients were kept on a calcium-constant diet.

### Materials and methods

### *Patients*

Consecutive patients with histologically proven adenomatous colorectal polyps were eligible. Polyps were found and excised during flexible endoscopy. Previous to polypectomy biopsies were taken for proliferation measurements as described below. Patients with other colonic abnormalities, especially colitis, members of families affected with familial adenomatous polyposis or hereditary nonpolyposis colon cancer (Lynch syndrome) and patients with previous colonic surgery were excluded. After histological verification of the adenomatous nature of the polyp(s), patients were informed about the study and were asked to participate. Seventeen patients, 11 men and six women, agreed to take part in the study. Their mean age was 56 (range 39-69) years. Informed consent was obtained and the study was approved by the medical ethical committee of the University Hospital of Groningen.

### *Epithelial cell proliferation*

Colonic epithelial cell proliferation was examined in the midsigmoid. To this end three biopsies were taken at 30 cm from the anal verge. Proliferation rate was determined by incubation of the biopsies with the thymidine analogue 5-bromodeoxyuridine (BrdU) and then visualising BrdU-labelled cells using immunohistochemistry as previously described (Welberg *et al.*, 1990). The proliferation rate was expressed as labelling index (LI) which is the number of labelled nuclei divided by the total number of nuclei times hundred (%). Only whole length cut crypts, containing at least 70 cells were used. Because length cut crypts are limited, we previously determined the minimal number of crypts necessary to obtain a reliable LI (Welberg *et al.*, 1990). Using the method of the running average we found this number to be 12. By dividing crypts in three compartments of equal length, LI of luminal, mid and basal compartments were determined. Slides were counted under blinded condition.

### *Study protocol*

After a dietary history taken by a dietician, patients were instructed to use a calcium constant diet, based on their own habitual diet, during the 13-week study period. Before the start of calcium supplementation patients used their diet during 1 week and collected faeces and urine during the last 24 h of this week. They then started to take 1.5 g $Ca^{2+}$ as calcium carbonate daily and continued this during 12 weeks. The calcium tablets were taken thrice a day with the meals.

Correspondence: J.H. Kleibeuker, Department of Internal Medicine, University Hospital, PO Box 30.001, 9700 RB Groningen, The Netherlands.

Received 29 June 1992; and in revised from 29 September 1992.

During the last week of the study period 24 h faeces and urine were collected again and biopsies were taken during flexible endoscopy. Calcium contents in faeces and urine were measured before and at the end of the intervention period as previously described (Van der Meer *et al.*, 1990*b*).

The first seven patients were asked to continue calcium carbonate after the 12 weeks intervention period, without adhering to the strictly calcium constant diet. These were biopsied again a year after start of calcium supplementation. Based on the initial results no further patients were requested to continue calcium after the first 12 weeks.

### *Statistical analysis*

Results of LI of total crypts and crypt compartments are presented as medians and ranges. Comparison of LI before and during calcium was made using the Wilcoxon test for paired results.

### Results

The calcium tablets were well tolerated by the patients. No mention was made about side effects, when asked for them.

The mean ( ± s.e.m.) number of crypts counted for the determination of the LI's was 17 ± 1, the mean number of nuclei counted was 1520 ± 63, and the mean number of BrdU-labelled nuclei was 134 ± 12 (Table I).

The median labelling index at initial biopsy in this group of polyp-patients was 6.1% (Figure 1). This is slightly but significantly higher than previously reported values from 10 subjects with a normal colon, who had a median LI of 5.4 (4.0–10.3)%, when determined by the same technique (Welberg *et al.*, 1990). During 12-weeks calcium supplementation the LI in the polyp patients increased to a median of 8.7% ($P<0.02$) (Figure 1). This effect was mainly due to an increase of LI in the basal part of the crypts. LI's in the mid and luminal crypt segments were not significantly affected by calcium (Table I).

The increase of epithelial proliferation rate during calcium sustained at longer follow-up. Median LI in the seven patients, biopsied after 1 year calcium supplementation, was 8.8 (5.9–11.2)%. When comparisons were made for this subgroup between LI at the start of the study and after 3 and 12 months of calcium, also an increase ($P<0.05$) was found, from 5.9 (5.4–8.3)% before calcium to 8.7 (3.9–14.2)% after 3 months and 8.8% after 12 months of calcium (Table I).

Calcium did not affect the length of colonic crypts. Mean ( ± s.d.) crypt length before calcium was 46 ± 6 cells and after 3 months calcium 47 ± 7 cells.

Compliance with diet and calcium tablets proved to be excellent. Total 24 h calcium excretion (mean ± s.d.) in faeces

**Table I** Mean (s.e.m.) numbers of crypts, nuclei and BrdU-labelled nuclei, counted in biopsies and median (range) labelling index in the whole crypt and in the luminal, mid and basal crypt compartments, before, after 12 weeks and after 1 year of calcium supplementation

| | *Before* $n = 17$ | *After 12 wks* $n = 17$ | *After 1 yr* $n = 7$ |
|---|---|---|---|
| Crypts | 17 | 16 | 18 |
| | (1) | (1) | (1) |
| All nuclei | 1479 | 1554 | 1534 |
| | (89) | (122) | (92) |
| BrdU-labelled | 112 | 156 | 134 |
| | (15) | (23) | (28) |
| LI total | 6.1% | 8.7%[b] | 8.8%[a] |
| | (4.5–17.3) | (3.9–19.5) | (5.9–11.2) |
| LI luminal | 0.4% | 0.9% | 0.4% |
| | (0.0–4.4) | (0.0–2.6) | (0.0–2.1) |
| LI mid | 7.2% | 9.0% | 8.5% |
| | (3.6–15.0) | (3.4–29.9) | (3.8–12.4) |
| LI basal | 11.9% | 16.4%[c] | 17.1%[b] |
| | (5.6–37.5) | (7.9–32.3) | (10.8–28.9) |

[a]$P<0.05$, [b]$P<0.02$, [c]$P<0.01$ *vs* before calcium.

**Figure 1** Individual and median (connected by underbroken line) labelling indexes (%) in 17 patients with adenomatous polyps before ($LI_1$) and after ($LI_2$) 12 weeks supplementation with 1.5 g $Ca^{2+}$.

and urine at the end of the first week of the study period, without calcium supplementation, was 38.5 ± 22.8 mmol ($n = 15$). During the last week of the supplementation period the 24 h excretion was 71.1 ± 32.4 mmol ($n = 15$). Since 35.5 mmol $Ca^{2+}$ was given each day, the recovery was 92%.

### Discussion

This study shows that oral calcium supplementation causes an increase of epithelial cell proliferation in biopsies from the sigmoid of patients with adenomatous polyps. This result is in contrast with those of several other groups of investigators (Lipkin *et al.*, 1985; Rozen *et al.*, 1989; Barsoum *et al.*, 1992; Wargovich *et al.*, 1992) who have shown a decrease of rectal epithelial proliferative activity during administration of calcium in similar doses. However, only the latter two studies were controlled, and in two other controlled studies (Gregoire *et al.*, 1989; Stern *et al.*, 1990) no effect of calcium supplementation was found. Our study also lacked a control group, but the strength of the results was increased by the finding that proliferation rate remained at the same elevated level during continued calcium supplementation. As opposed to other studies our patients were instructed to keep a calcium-constant diet which was well complied with. Thus the effects on colonic cell proliferation can be ascribed to the supplemental calcium and were not due to some unforeseen modification in the intake of dietary calcium.

Lipkin *et al.* (1989) recognised in a group of subjects at risk for familial colon cancer, so called responders and non-responders to calcium. The former proved to have a higher mean initial labelling index compared to the latter. This suggests that the degree of hyperproliferation may affect the effects of calcium. We could not confirm this in our patients.

Nearly all showed an increase of LI during calcium supplementation, also those, except two, with a LI in the higher range.

The supposition about the beneficial effects of calcium on the colon has been supported by the results of several epidemiologic studies (Garland *et al.*, 1985; Kune *et al.*, 1987; Slattery *et al.*, 1988). However, this effect could not be found in all studies (Heilbrun *et al.*, 1986; Negri *et al.*, 1990). Differences in dietary sources of calcium and other confounding factors in the nutritional patterns of the populations studied might account for the observed differences. The same may be true for the discrepancy between our data and those of others in regard to the epithelial proliferation rate. It is noteworthy in this respect that our patients probably had a much higher dietary calcium intake than the subjects studied by Lipkin *et al.* (1985) and by Wargovich *et al.* (1992). Whereas the latter ones had a daily intake of around 700 mg, our patients had a 24 h calcium excretion of about 1,600 mg. Although this value probably slightly overestimates 24 h intake, due to the fact that not all patients had daily defecation, a clear difference seems to exist. It is therefore mandatory that in future intervention studies attention will be paid to the composition of the diet.

An important and perhaps essential difference between this study and others is the site in the intestine for determination of the proliferation rate. In the studies published so far biopsies were taken from the rectum (Lipkin & Newmark, 1985; Rozen *et al.*, 1989; Gregoire *et al.*, 1989; Stern *et al.*, 1990; Barsoum *et al.*, 1992; Wargovich *et al.*, 1992), whereas we took them from the sigmoid. This may have had important implications for the effect of calcium. From epidemiological surveys it has become apparent that there are differences in the association of dietary factors with colon cancer on the one hand and with rectal cancer on the other (Ziegler *et al.*, 1986). Such a difference may also exist for calcium. However, the previously mentioned epidemiological studies suggest that the protective effect of calcium is not limited to the rectum but implies the whole large bowel (Garland *et al.*, 1985; Kune *et al.*, 1987; Slattery *et al.*, 1988).

It should thus be considered whether the discrepancy with other studies is due to specific untoward effects of the mode of calcium intervention on the sigmoid epithelium. We have previously shown favourable effects of calcium carbonate on duodenal bile acid composition (Van der Meer *et al.*, 1990*b*) and on some characteristics of the faecal water, including its cytotoxicity *in vitro* (Van der Meer *et al.*, 1990*a*). On the other hand, calcium carbonate is generally stated to be a constipating agent. This quality could lead to a prolongation of colonic transit time and thereby of the exposure time of the colonic epithelium to intestinal contents. This may result in an increase in epithelial damage by toxic components despite their lower concentrations in the faecal material. Since under normal circumstances the rectum is only filled shortly before defecation (McNeil *et al.*, 1981), the favourable change in faecal composition by calcium can have its beneficial effects on rectal epithelium, whereas the epithelium of the distal colon can be affected untowardly by the prolonged residence of faeces there.

Faecal pH increases during calcium carbonate administration by about 0.3 pH-units (Van der Meer *et al.*, 1990*b*). A high faecal pH has been hypothesised to be associated with an increased risk for colonic cancer (Thornton, 1981) and there is epidemiologic evidence to support this (Walker *et al.*, 1986). It is questionable however, whether such small modification of faecal pH in the range around 6.5, as caused by calcium carbonate, might have implications for the colonic epithelium.

It is concluded, that there is no single explanation for the observed differences between the responses of the epithelium to calcium in the sigmoid and the rectum. Nevertheless our observations may have several implications for current and future investigations on the possible role of calcium in the prevention of colon cancer. As previously mentioned, in most studies performed so far biopsies have been taken from the rectal mucosa. From our results it seems mandatory to modify this practice and to take biopsies also from more proximal parts of the colon, including the sigmoid. Furthermore, calcium carbonate may not be the right formula for intervention and it should be considered to study other calcium compounds or to combine calcium with other measures, for example fibre. Recently some large-scale intervention studies with calcium have been launched in several countries. A prudent approach with respect to these studies seems to be warranted in view of our results. More data about the effects of calcium on the epithelium of the whole large bowel and about the mechanisms through which these effects are being mediated, should be collected.

This study was supported by a grant from the Dutch Cancer Society (GUKC 89-08). The authors thank N. Zwart for excellent technical assistance.

### References

BARSOUM, G.H., HENDRICKSE, C., WINSLET, M.C., YOUNGS, D., DONOVAN, I.A., NEOPTOLEMOS, J.P. & KEIGHLEY, M.R.B. (1992). Reduction of mucosal crypt cell proliferation in patients with colorectal adenomatous polyps by dietary calcium supplementation. *Br. J. Surg.*, **79**, 581–583.

GARLAND, C., BARRET-CONNER, E., ROSSOF, A.H. SHEKELLE, R.B., CRIQUI, M.H. & PAUL, O. (1985). Dietary vitamin D and calcium and risk of colorectal cancer: a 19-year prospective study in men. *Lancet*, **11**, 307–309.

GREGOIRE, R.C., STERN, H.S., YEUNG, K.S., STADLER, J. LANGLEY, S., FURRER, R. & BRUCE, W.R. (1989). Effect of calcium supplementation on mucosal cell proliferation in high risk patients for colon cancer. *Gut*, **30**, 376–382.

HEILBRUN, L.K., HANKIN, J.H., NOMURA, A.M.Y. & STEMMERMAN, G.N. (1986). Colon cancer and dietary fat, phosphorus, and calcium in Hawaiian-Japanese men. *Am. J. Clin. Nutr.*, **43**, 306–309.

KUNE, S., KUNE, G.A. & WATSON, L.F. (1987). Case-control study of dietary etiological factors: the Melbourne colorectal cancer study. *Nutr. Cancer*, **9**, 21–42.

LIPKIN, M., FRIEDMAN, E., WINAWER, S.J. & NEWMARK, H. (1989). Colonic epithelial cell proliferation in responders and nonresponders to supplemental dietary calcium. *Cancer Res.*, **49**, 248–254.

LIPKIN, M. & NEWMARK, H. (1985). Effect of added dietary calcium on colonic epithelial-cell proliferation in subjects at high risk for familial colonic cancer. *N. Engl. J. Med.*, **313**, 1381–1384.

MCNEIL, N.I., RAMPTON, D.S. & PHIL, D. (1981). Is the rectum usually empty? A quantitative study in subjects with and without diarrhea. *Dis. Colon Rectum*, **24**, 596–599.

NEGRI, E., LA VECCHIA, C., D'AVANZO, B. & FRANCESCHI, S. (1990). Calcium, dairy products, and colorectal cancer. *Nutr. Cancer*, **13**, 255–262.

NEWMARK, H.L., WARGOVICH, M.J. & BRUCE, W.R. (1984). Colon cancer and dietary fat, phosphate, and calcium: a hypothesis. *J. Natl Cancer Inst.*, **72**, 1323–1325.

RISIO, M., LIPKIN, M., CANDELARESI, G.-L., BERTONE, A., COVERLIZZA, S. & ROSSINI, F.P. (1991). Correlations between rectal mucosa cell proliferation and the clinical and pathological features of nonfamilial neoplasia of the large intestine. *Cancer Res.*, **51**, 1917–1921.

ROSEN, P., FIREMAN, Z., FINE, N., WAX, Y. & RON, E. (1989). Oral calcium suppresses increased rectal epithelial proliferation of persons at risk of colorectal cancer. *Gut*, **30**, 650–655.

SCALMATI, A., RONCUCCI, L., GHIDINI, G., BIASCO, G. & PONZ DE LEON, M. (1990). Epithelial cell kinetics in the remaining colorectal mucosa after surgery for cancer of the large bowel. *Cancer Res.*, **50**, 7937–7941.

SLATTERY, M.L., SORENSEN, A.W. & FORD, M.H. (1988). Dietary calcium intake as a mitigating factor in colon cancer. *Am. J. Epidemiol.*, **128**, 504–514.

STERN, H.S., GREGOIRE, R.C., KASHTAN, H., STADLER, J. & BRUCE, R.W. (1990). Long-term effects of dietary calcium on risk markers for colon cancer in patients with familial polyposis. *Surgery*, **108**, 528–533.

TERPSTRA, O.T., VAN BLANKENSTEIN, M., DEES, J. & EILERS, G.A.M. (1987). Abnormal pattern of cell proliferation in the entire colonic mucosa of patients with colon adenoma or cancer. *Gastroenterology*, **92**, 704–708.

THORNTON, J.R. (1981). High colonic pH promotes colorectal cancer. *Lancet*, **1**, 1081–1082.

VAN DER MEER, R., LAPRÉ, J.A., KLEIBEUKER, J.H., DE VRIES, E.G.E. & DE VRIES, H.T. (1990*a*). Effects of supplemental dietary calcium on composition and cytotoxicity of fecal water. *Gastroenterology*, **98**, A317.

VAN DER MEER, R., WELBERG, J.W.M., KUIPERS, F., KLEIBEUKER, J.H., MULDER, N.H., TERMONT, D.S.M.L., VONK, R.J. DE VRIES, H.T. & DE VRIES, E.G.E. (1990*b*). Effects of supplemental dietary calcium on the intestinal association of calcium, phosphate, and bile acids. *Gastroenterology*, **99**, 1653–1659.

WALKER, A.R.P., WALKER, B.F. & WALKER, A.J. (1986). Faecal pH, dietary fibre intake, and proneness to colon cancer in four South African populations. *Br. J. Cancer*, **53**, 489–495.

WARGOVICH, M.J., ISBELL, G., SHABOT, M., WINN, R., LANZA, F., HOCHMAN, L., LARSON, E., LYNCH, P., ROUBEIN, L. & LEVIN, B. (1992). Calcium supplementation decreases rectal epithelial cell proliferation in subjects with sporadic adenoma. *Gastroenterology*, **103**, 92–97.

WEISBURGER, J.H. & WYNDER, E.L. (1987). Etiology of colorectal cancer with emphasis on mechanism of action and prevention. In *Important Advances in Oncology*. DeVita, V.T. Jr, Hellman, S. & Rosenberg, S.A. (eds) pp. 197–220. J.B. Lippincott: Philadelphia.

WELBERG, J.W.M., DE VRIES, E.G.E., HARDONK, M.J., MULDER, N.H., HARMS, G., GROND, A.J., ZWART, N., KOUDSTAAL, J. DE LEY, L. & KLEIBEUKER, J.H. (1990). Proliferation rate of colonic mucosa in normal subjects and patients with colonic neoplasms: a refined immunohistochemical method. *J. Clin. Pathol.*, **43**, 453–456.

ZIEGLER, R.G., DEVESA, S.S. & FRAUMENI, J.F. Jr (1986). Epidemiologic patterns of colorectal cancer. In *Important Advances in Oncology 1986*, DeVita, V.T. Jr, Hellman, S. & Rosenberg, S.A. (eds) pp. 209–232. J.B. Lippincott: Philadelphia.